# Torsional Properties of Bundles with Randomly Packed Carbon Nanotubes

**DOI:** 10.3390/nano12050760

**Published:** 2022-02-24

**Authors:** Hanqing Wei, Heidi Zhi Jin Ting, Yongji Gong, Chaofeng Lü, Olga E. Glukhova, Haifei Zhan

**Affiliations:** 1 Department of Civil Engineering, Zhejiang University, Hangzhou 310058, China; hanqing.wei@outlook.com (H.W.); lucf@zju.edu.cn (C.L.); 2 School of Mechanical, Medical and Process Engineering, Queensland University of Technology (QUT), Brisbane, QLD 4001, Australia; heidizhijin.ting@connect.qut.edu.au; 3 School of Materials Science and Engineering, Beihang University, Beijing 100191, China; yongjigong@buaa.edu.cn; 4 Soft Matter Research Center, Zhejiang University, Hangzhou 310027, China; 5 Faculty of Mechanical Engineering & Mechanics, Ningbo University, Ningbo 315211, China; 6 Department of Physics, Saratov State University, Astrakhanskaya 83, 410012 Saratov, Russia; 7 Institute for Bionic Technologies and Engineering, I.M. Sechenov First Moscow State Medical University (Sechenov University), 119991 Saratov, Russia; glukhovaoe@info.sgu.ru

**Keywords:** carbon nanotube bundle, torsion, random packing, molecular dynamics simulation

## Abstract

Carbon nanotube (CNT) bundles/fibers possess promising applications in broad fields, such as artificial muscles and flexible electronics, due to their excellent mechanical properties. The as-prepared CNT bundles contain complex structural features (e.g., different alignments and components), which makes it challenging to predict their mechanical performance. Through in silico studies, this work assessed the torsional performance of CNT bundles with randomly packed CNTs. It is found that CNT bundles with varying constituent CNTs in terms of chirality and diameter exhibit remarkably different torsional properties. Specifically, CNT bundles consisting of CNTs with a relatively large diameter ratio possess lower gravimetric energy density and elastic limit than their counterpart with a small diameter ratio. More importantly, CNT bundles with the same constituent CNTs but different packing morphologies can yield strong variation in their torsional properties, e.g., up to 30%, 16% and 19% difference in terms of gravimetric energy density, elastic limit and elastic constants, respectively. In addition, the separate fracture of the inner and outer walls of double-walled CNTs is found to suppress the gravimetric energy density and elastic limit of their corresponding bundles. These findings partially explain why the experimentally measured mechanical properties of CNT bundles vary from each other, which could benefit the design and fabrication of high-performance CNT bundles.

## 1. Introduction

Carbon nanotube (CNT) bundles/fibers have received enormous interest from both scientific and engineering communities, due to their promising applications as mechanical energy storage media [1], energy harvesters [2], promising building blocks for artificial muscles [3,4] and flexible electronics [5,6]. There are several types of CNT bundles/fibers being studied, including CNT fibers/bundles [7,8,9,10], vertically aligned carbon nanotube arrays [11,12] and yarns [13,14], which are fabricated through either a spinning [15] or twisting/rolling technique [16]. With the continuous optimization of synthesis technology and methods, ultra-straight CNT bundles with a tensile strength of over 80 GPa have been prepared successfully [17], based on ultralong defect-free CNTs. In experiments, the size and type of individual CNT can be controlled during the synthetization process [18]; while it is still challenging to prepare CNT bundles with uniform constituent CNTs and most experimentally studied CNT bundles comprise of different kinds of CNTs [17,19]. As a result, the experimentally measured elastic properties of CNT bundles are usually not consistent and show a relatively large variation. For example, their Young’s modulus ranges from 70 to 350 GPa [20,21,22].

To tackle the challenges in precisely controlling the properties of CNT bundles, molecular dynamics simulation that is based on the atomistic configurations of CNT becomes a powerful tool. There are extensive atomistic works exploring the mechanical properties of individual CNTs and CNT bundles. It is found that the torsional behavior of a single-walled carbon nanotube (SWNT) is dependent on not only the tube chirality but also on the loading direction [23]. Under torsional deformation, CNT is prone to buckling due to its hollow tubular geometry [24,25,26]. The critical buckling load for both zigzag and armchair CNTs increases as the tube diameter increases [27]. Buckling of constituent CNT introduces structural instability, while it may also be beneficial for certain applications as the appearance of buckling allows large deformation [28,29]. Furthermore, it is found that the shear modulus of zigzag CNT is higher than that of armchair CNT [30], and the zigzag structure shows superior performance against torsional buckling than that of the armchair structure [31]. Previous works report that when the tube diameter is less than 10, the shear modulus of the CNT bundle is linearly related with the CNT diameter [32], and the increase of constituent CNTs in the bundle structure will lead to a decrease in the strain energy density under torsion [33]. In addition to full atomic studies, a coarse-grained model has also been employed to investigate the mechanical properties and microstructural evolution of CNT fibers under twisting [13,34]. It is found that the coupling effect of bending, torsional, tensile and compressive deformations play a key role in the mechanical energy stored within twisted SWNT-based bundles [33,35].

Recently, a CNT bundle structure constructed of a mixture of SWNTs and multi-walled CNTs, and impacts of factors, such as tube density, tube distribution, metallic tube ratio and bundle dimensions was discussed to study the kinetic inductance performance [36]. Studies reveal that the stacking morphology will influence the tensile properties of double-walled CNT (DWNT) bundles [37]. Thus far, almost all studies on CNT bundles under torsion have considered a bundle structure with a uniform constituent which, however, is not the case in experimentally prepared samples. As such, it is of great interest to assess how the bundle will perform when it has a randomly packed configuration, which becomes the focus of this work. Through molecular dynamics simulations, it is found that the non-uniformity of the chirality, the diameter ratio (ratio between the maximum and minimum constituent CNTs) and the mass density of bundle structures (bundle structures with a similar effective diameter but different numbers of CNTs) will significantly impact the torsional properties of the bundle structure.

## 2. Methods

### 2.1. Bundle Packing Method

**Figure 1:**
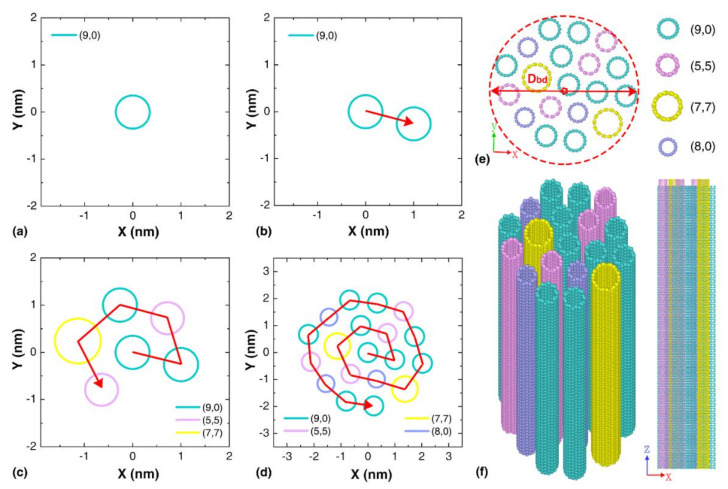
Schematic view of the packing method. The general procedure of the random circle packing method, including: (**a**) initialization; (**b**) adding the second CNT; (**c**) locating the sixth CNT; and (**d**) locating the nineteenth CNT. Each ring represents the cross-section of a CNT. A presentative CNT bundle containing 19 SWNTs: (**e**) the cross-sectional view, and (**f**) the side view.

The circle packing concept [38] was implemented to pack nanotubes with different diameters to form a cylindrical CNT array, mimicking the cross-sectional structure of the CNT yarn observed in experiments [39,40]. Experiments show that CNTs tend to be self-assembled into circular cross-section (consisting of large diameter CNTs) [37] and regular polygon cross-section (consisting of CNTs with a small diameter) [33] structures. A house-code was developed based on the population-based solution [41], which randomly selects CNTs from a sample pool and packs them axially together to form a bundle. The sample pool contains SWNTs with different zigzag and armchair structures. Specifically, the first CNT was arranged in the center of the bundle as shown in Figure 1a, followed by subsequent CNTs being arranged in a spiral that is tangent to the surrounding CNTs. Positioning of subsequent CNTs is determined by marking the tangent of the surrounding CNTs and measuring the radial distance between the tangents to determine the center of the subsequent CNT, as schematically shown in Figure 1b–d. This method yields a relatively compact arrangement of CNTs in the bundle with minimal space variance between CNTs. The initial inter-tubular distance of 3.4 Å was considered, which is the same as the graphitic interlayer spacing distance [22,42]. Figure 1e–f shows atomic configurations of a representative CNT bundle composed of a total of 19 nanotubes with indices (5,5), (7,7), (8,0) and (9,0). The effective diameter of the CNT-based bundles is referred as *D_bd_*.

### 2.2. Simulation Details

The molecular dynamic simulations were performed via Large-scale Atomic/Molecular Massively Parallel Simulator (LAMMPS) [43]. The AIREBO potential [44] was employed to describe the carbon–carbon interactions, which has been widely used to describe the mechanical behaviors of carbon nanostructures, such as graphene, CNTs and CNT bundle [22,45]. The cut-off distance for the switching function was modified as 2.0 Å to avoid the non-physical high tensile stress under bond stretching [46,47,48,49]. Before the torsional testing, the CNT bundles were optimized by the conjugate gradient minimization method and then equilibrated using the Nosé–Hoover thermostat [50] for 1200 ps to obtain a state of equilibrium under canonical ensemble. Periodic and free boundary conditions were applied along the axial and radial directions, respectively. Then, the periodic boundary conditions were switched off. A couple of rotational loads were imposed simultaneously on both ends of the CNT bundle in opposite direction with a period of 6000 ps, corresponding to a rotational speed of 2π/12,000 rad/ps, which is the same as applied in previous work [33]. A small timestep of 0.5 fs was applied in the relaxation and mechanical testing process. The temperature of the CNT bundles was maintained at 1 K during the simulation to limit the influence of thermal fluctuations.

During the simulation, virial stress was calculated, which is defined as [51]:

(1) $$\pi_{i}^{\alpha \beta }=\frac{1}{\bar{\omega_{t}}}\left\{-m_{i}v_{{}_{i}}^{\alpha }v_{{}_{i}}^{\beta }\right.+\left.\frac{1}{2}\sum_{j\neq i}F_{ij}^{\alpha }r_{ij}^{\beta }\right\}$$

where ${\bar{\omega }}_{t}$ represents the effective volume of atom *i* and $\Omega ={\bar{\omega }}_{t}$; $m_{i}$ and $v_{i}$ are the mass and velocity of atom i; $F_{ij}$ and $r_{ij}$ are the force and distance between atoms i and j; and the indices α and β represent the Cartesian components. Considering the sophisticated stress status that the atoms experience in the deformed carbon nanotube bundle, the atomic Von Mises stress was also calculated based on the atomic virial stress.

Based on Hooke’s law, the total strain energy for a twisted bundle structure with different numbers of CNTs can be expressed as $\Delta E_{tot}=\sum \left(\Delta E_{t}+\Delta E_{s}+\Delta E_{b}+\Delta E_{c,ij}\right)$ [33,52,53]. Considering the total mass of the bundle as *M*, the strain energy density $\Delta E_{tot}/M$ is derived as:

(2) $$\Delta E_{\mathrm{tot}}/M=\frac{1}{n}\left[nk_{t}\varepsilon_{t}^{2}+k_{s}\sum_{i=1}^{n}\varepsilon_{s,i}^{2}+k_{b}\sum_{i=1}^{n}\varepsilon_{b,i}^{2}+k_{c}\sum_{i=1}^{n}\varepsilon_{c,ij}^{2}\right]$$

Here, the dimensionless torsional strain is defined as $\varepsilon_{t}=\varphi D_{0}/l_{0}$, with $D_{0}$ and $l_{0}$ as the equivalent diameter and length of the sample, and φ as the twist angle. *n* is the filament number, *m* is the mass of each filament. The tensile strain set as $\varepsilon_{s,i}=\sqrt{1+{\left(\rho_{i}\varphi /l_{0}\right)}^{2}}-1$. The bending strain set as $\varepsilon_{b,i}=D_{0}/R$, and $R_{i}=\rho_{i}\left[1+{\left(l_{0}/\rho_{i}\varphi \right)}^{2}\right]$ [54], $\rho_{i}$ is the coil radius, which is the equilibrium separation distance between the filament axis and the bundle axis. The compressive strain was calculated from $\varepsilon_{c,ij}=1-d_{0}/d_{ij}$. For simplicity, each filament was considered to have the same equilibrium distance $d_{0}$ to its closest neighbors, and $d_{ij}$ is the closest neighbor inter-tubular distance.

## 3. Results and Discussions

### 3.1. Torsional Properties of Individual SWNTs

Before exploring the torsional performance of the bundle structure, we compare the torsional properties of the different armchair and zigzag SWNTs, as summarized in Table 1. For discussion convenience, the armchair and zigzag SWNTs are denoted as A_m_ and Z_n_, respectively, with *m* and *n* representing the CNT index number, respectively. The detailed simulation results including the strain energy curves and the atomic configurations are provided in Supporting Information Figure S1. According to Table 1, the gravimetric energy density of the SWNTs ranges from 2.0–3.15 MJ/kg and the structure is able to achieve a relatively large torsional angle of 12.2–18.0 rad (or 0.5–0.7 rad/nm) before any bond breakage, which aligns well with previous work [33]. Here, the gravimetric energy density ($E_{lim}$) refers to the maximum strain energy density ($\Delta E_{max}/M$) that the SWNTs can achieve before fracture, and the corresponding dimensionless torsional strain is referred to as the torsional elastic limit ($\varepsilon_{lim}$). M is the total mass of the CNT, and the strain energy is computed from $\Delta E=E_{t}-E_{0}$, with $E_{t}$ and $E_{0}$ representing the potential energy of the strained and initial bundle.

According to continuum mechanics [33,55], the torsional strain energy is a parabolic function of the torsional strain in the linear elastic deformation regime, i.e., $\Delta E/M=k_{T}\varepsilon_{T}^{2}/2$. From the atomic configurations at different deformation stages, SWNTs experience torsion-induced helical buckling, torsional buckling [56,57], and collapsed wall induced folding [58,59] when the torsional strain increases. The occurrence of buckling or flattening for SWNTs with a relatively large diameter deviates the strain energy curve from the ideal parabolic relationship (see Supporting Information Figure S2). Meanwhile, the buckling or flattening phenomenon relies on the geometrical feature (i.e., length and diameter) of the SWNT, and thus, the fracture behavior of different SWNTs varies from each other. Additionally, the elastic constant increases generally when the diameter of CNT increases, which is expected due to the fact that a larger diameter means a larger polar moment of inertia and more atoms involved in the deformation. Generally, zigzag CNTs experience early fractures than the armchair ones with similar diameters, this is consistent with the fracture mechanisms of graphene reported in the literature [60], i.e., the crack propagates preferentially along the zigzag direction. Here, the elastic constant is fitted from the strain energy profile with a torsional strain within ~0.05 (i.e., in the linear elastic regime).

### 3.2. Bundle Structure Consists of SWNTs with Different Chirality

With the above understanding, we then examine the torsional properties of bundles consisting of SWNTs with a similar diameter. That is, the diameter ratio (η) approximates to 1, which is defined as $\eta =d_{l}/d_{s}$, where $d_{l}$ and $d_{s}$ are the diameter of the largest and smallest constituent CNTs, respectively. Different armchairs and zigzag SWNTs are selected to form the bundle structure, and a filament number of 19 is considered in comparison with the bundle with 19 uniform constituent SWNTs (which forms a uniform hexagonal cross-section). As listed in Table 2, five groups of bundles are considered with an overall diameter in the range from 4.8 to 12.0 nm, and each group contains three models, including model $Z_{n}^{19}$—consists of only zigzag (n,0) SWNTs, model $A_{m}^{19}$—consists of only armchair (m,m) SWNTs, and model $A_{m}^{i}Z_{n}^{j}$—consists of a mixture of armchair and zigzag SWNTs. Here, i and j represent the number of SWNTs and i+j=19. In each group, the effective diameter of the bundle structures ($D_{bd}$) is similar, which is estimated by selecting the initial CNT during the packing process as the geometrical center of the bundle (Figure 1a). The total cross-sectional area ($S_{bd}$) of each bundle is estimated from $S_{bd}=\sum_{i}s_{i}$, with $s_{i}$ as the cross-sectional area of the ith SWNT. The cross-sectional views of these models are presented in Supporting Information Figure S3. In theory, each constituent SWNT bundle experiences torsion, tension, bending and radial compression during the torsional deformation. Previous works [33,35] suggest that the contribution from the tensile deformation surpasses the contributions from the other three deformation modes in the bundle structure when its filament number is over seven. Obviously, the constituent CNTs on the outer annulus with the largest coil radius experiences the highest tensile strain. That is, the outer layer SWNTs will fracture first during torsion.

According to Table 2, the bundle structure consists of pure zigzag SWNTs usually have a smaller elastic limit than its counterpart consisting of pure armchair SWNTs. Such an observation is in line with the results shown in Table 1, where the zigzag SWNTs normally experience earlier fracturing than their armchair counterparts with a similar diameter. In comparison, the mixed bundle exhibits a medium torsional property in groups 2–4 with η≤1.03, which is higher than the bundle consisting of pure zigzag SWNTs, but lower than the bundle consisting of pure armchair SWNTs. For instance, in group 2, the $A_{7}^{8}Z_{12}^{11}$ bundle exhibits a gravimetric energy density of 558.18 kJ/kg, about 10% higher than the pure $Z_{12}^{19}$ bundle (508.77 kJ/kg) but much smaller than the pure $A_{7}^{19}$ bundle (642.94 kJ/kg). However, when the diameter ratio is relatively large in group 1 and group 5, the mixed bundle is found to exhibit poorer torsional performance than its counterpart either consisting of pure zigzag SWNTs or armchair SWNTs. The relative difference of the gravimetric energy density $\zeta_{E}=\left(E_{lim}^{max}-E_{lim}^{min}\right)/E_{lim}^{max}$ in each group is in the range of 20–28%. Here, $E_{lim}^{max}$ and $E_{lim}^{min}$ represent the largest and smallest gravimetric energy density in each group. Similarly, the relative difference of the elastic limit $\zeta_{\varepsilon }$ is calculated for each group, which is also relatively large (between 8% and 20%). Different from the gravimetric energy density/elastic limit, the elastic constant for the bundles in each group is quite similar with a minor relative difference of less than 10%.

**Figure 2:**
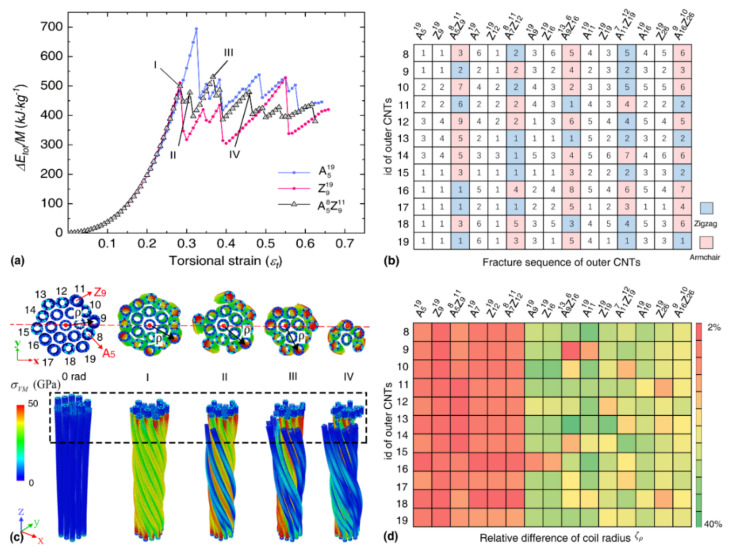
The torsional deformation for SWNT-based bundles with different chirality. (**a**) The torsional strain energy density as a function of the dimensionless torsional strain for $A_{5}^{19},$ $Z_{9}^{19}$ and $A_{5}^{8}Z_{9}^{11}$ bundle; (**b**) Fracture sequence of the outer CNTs for different bundles; the marked pink and blue regions indicated the armchair and zigzag CNTs, respectively, and the number in the grid indicates the observed fracture sequence; (**c**) Atomic configurations at different deformation stages for $A_{5}^{8}Z_{9}^{11}$ bundle. I-VI correspond to the torsional deformation stages of strain energy density curves in Figure 2a. Top panels are the cross-sectional views of the bundles. The atomic configurations only show a segment (~6 nm) that close to the fracture region of the sample, and only unfractured CNTs are visualized. The red dashed lines highlight the center of the applied torsional load. Atoms are colored according to the atomic Von Mises (VM) stress. Bottom panels are the side views; and (**d**) The relative difference of the coil radius ($\zeta_{\rho }$) for each outer CNT between the initial state and the elastic limit stage.

Figure 2a compares the torsional strain energy curves for the bundles in group 1 with an effective diameter of about 4.80 nm. It is seen that the strain energy profiles for different bundles are nearly overlapped with each other in the elastic regime before fracture, suggesting a similar elastic constant for different bundles observed in Table 2. After fracture, multiple strain energy drop events are observed, which are induced by the multiple-step fracture of SWNTs in the bundle structure during torsion. For the highlighted peaks in the torsional strain energy curve of $A_{5}^{8}Z_{9}^{11}$ bundle in Figure 2a, the corresponding tensile strain ($\varepsilon_{str,i}$) of the outer CNTs are $\varepsilon_{I}=0.05$, $\varepsilon_{II}=0.06$, $\varepsilon_{III}=0.08$ and $\varepsilon_{IV}=0.09$, respectively. For the mixed bundle structure, the outer layer zigzag CNTs fracture earlier than the armchair CNTs as presented in Figure 2b, which aligns with the tensile properties of CNTs (i.e., armchair CNT has a larger fracture strain). Note, the fracture sequence of bundle-$A_{5}^{8}Z_{9}^{11}$ (the strain energy curve experiences nine sudden drops) is the highest, which means that the stress transmission efficiency between CNTs is relative worse than other CNT bundles. This observation aligns with the relatively low gravimetric energy density of bundle-$A_{5}^{8}Z_{9}^{11}$ in group 1 (see Table 2). As evidenced in Figure 2c (upper panel), the outer layer SWNTs suffers from higher stress than the inner SWNTs, and thus, they experience fracture first. Since the diameter of the constituent SWNTs in the $A_{5}^{8}Z_{9}^{11}$ bundle is relatively small, no buckling or flattening phenomenon is observed during torsion. The fracture event releases part of the strain energy, which will change the morphology of the cross-section and eventually affect the deformation behavior of the unfractured SWNTs. As shown in Figure 2d, the relative difference of the coil radius ($\zeta_{\rho }$) for each outer CNT between the initial state and the elastic limit stage increases from 2% to 40% when the CNT diameter increases. Here, $\zeta_{\rho }$ is calculated from $\zeta_{\rho }=\left(\rho_{ini}-\rho_{lim}\right)/\rho_{ini}$, $\rho_{ini}$ and $\rho_{lim}$ represent the coil radius of the filaments at the initial stage and elastic limit stage, respectively. As the diameter of the CNTs increases, $\rho_{ini}$ of the bundles increases uniformly from 17 Å to 55 Å, and $\rho_{lim}$ varies with the cross-section of each filament. Overall, the above results signify that the bundles consisting of SWNTs with a similar diameter behave similarly in the elastic regime, while the elastic limit, as well as the gravimetric energy density, differ from each other due to the variation in stress distribution.

### 3.3. Bundle Structure Consists of SWNTs with Different Diameter Ratios

The above results indicate that the diameter ratio could be an influencing factor that affects the torsional behaviors of the bundle structure. To probe the potential influence, we compare the torsional properties of the bundle structures consisting of CNTs with different diameters, while keeping their overall diameters as a constant. For simplicity, the bundle constructed from two types of CNTs is adopted, and three groups of bundle structures are considered as listed in Table 3. The bundles constructed from CNTs with a smaller η normally have a higher gravimetric energy density and elastic limit, but a smaller torsional elastic constant. Such a trend is observed in all three groups. For instance, the elastic limit for $Z_{5}^{7}Z_{8}^{12}$ bundle with η = 1.60 is about 0.317, which is about 8% smaller than its counterpart $Z_{6}^{8}Z_{7}^{11}$ (η = 1.17). The largest difference is found for the bundle with an overall diameter of 6.25 nm, where the $Z_{12}^{9}Z_{13}^{10}$ bundle with a diameter ratio of 1.24 exhibits a gravimetric energy density of 455.50 kJ/kg, around 17.0% higher than its counterpart $Z_{10}^{10}Z_{15}^{9}$ (about 377.66 kJ/kg). Despite that, the relative difference of the elastic limit or gravimetric energy density between different bundles does not exhibit a direct relationship with the diameter ratio. Different from the results in Table 2, the difference in the elastic constant for the bundles in each group is much larger. For example, the elastic constant of the $A_{7}^{7}Z_{12}^{12}$ (η = 1.71) bundle is about 25% larger than its counterpart $Z_{9}^{7}Z_{10}^{12}$ (η = 1.11) in group 2 with an effective diameter of 5.08 nm. Overall, these results signify that a larger diameter ratio introduces a negative effect to the gravimetric energy density (elastic limit) of the bundle structure, but increases their elastic constant.

Unlike the bundles with uniform constituent CNTs or constituent CNTs with a similar diameter (i.e., η≈1), the coil radius of the outer CNTs varies remarkably from each other when η is relatively large. FromFigure 3a–b, it is obvious that the twisting center of bundles with a relatively large η is obviously offset from the geometry center, which is responsible for the lower gravimetric energy density and elastic limit compared with the bundles with smaller η. Several CNTs in the bundles with lower η tend to fracture at the same time, which thus, results in fewer fracture steps than their counterpart with larger diameter ratios (Supporting Information Figure S4). Comparing the strain energy profiles for bundles in group 1, from which an obvious gap is observed between the two curves before fracture, suggests different elastic deformation behaviors of the bundle structure. Similar as observed previously, multiple strain energy drop events are observed, which are caused by the multiple step fracture of the constituent CNTs under torsion.

**Figure 4:**
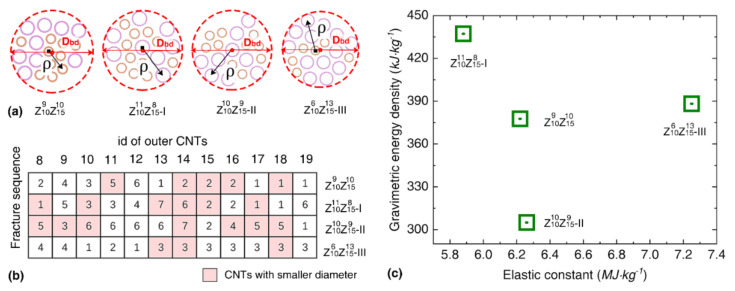
The comparisons of the torsional properties of bundles with same constituent SWNTs but different packing morphologies. (**a**) Different packing mode of bundle structures with a diameter ratio of 1.5. (**b**) Inset shows the fracture sequence of the outer CNTs. The pink grids refer to the CNTs with smaller diameter and the number in the grid represents the fracture sequence of the corresponding CNT. (**c**) The relation between the gravimetric energy density and the elastic constant of the bundles with different packing morphologies.

Since the coil radius of the constituent CNTs will affect the stress distribution, we compare the torsional behavior of bundles with the same consistent CNTs (diameter ratio of 1.5) but different packing morphologies, as presented in Figure 4a. By numbering the fracture sequence of the outer SWNTs (Figure 4b), we find that the bundle $Z_{10}^{6}Z_{15}^{13}$-III with a smaller fracture sequence number possesses a higher elastic constant. The coil radius of the bundles and the corresponding strain energy profiles are presented in Supporting Information Figure S5. It is found that the bundle with a larger coil radius and higher variance of coil radius of outer CNTs tend to exhibit a smaller gravimetric energy density, while the elastic constant does not show a clear relationship with the coil radius of the outer CNTs. As compared in Figure 4c, the gravimetric energy density changes from 305.0 to 437.0 kJ/kg (elastic limit ranges from 0.175–0.208), and the elastic constant varies between 5.88 and 7.25 MJ/kg. Overall, the packing morphology is found to induce 30%, 16% and 19% difference to the gravimetric energy density, elastic limit and elastic constant of the bundle structure.

### 3.4. Bundle Structure Consists of SWNTs with Different Mass Density

Recall Table 1, the bundles with the same effective diameter but containing different consistent CNTs possess a different total number of atoms for the same given length. In other words, they have different mass densities, and the bundles constructed from large CNTs have a smaller mass density. For illustration, we compare the torsional behaviors of another three groups of bundle structures, and each group contains two bundles with the same effective diameter, namely a bundle with seven large CNTs and a bundle with 19 small CNTs (Table 4). The strain energy curves of these bundles are listed in Supporting Information Figure S6. As expected, the bundle with more CNTs has a higher gravimetric energy density compared with the sample with fewer CNTs, and this difference increases when the effective diameter of the bundle increases. Such trend is observed in groups 1 and 2 and an adverse trend is observed in group 3, which can be explained from the fracture mode of the bundle as discussed below. Different from the gravimetric energy density or elastic limit, the elastic constant increases uniformly when the effective diameter increases in each group and the relative difference of the elastic constant decreases from 73% to 53%. We should note that the volumetric energy density shows a different pattern from that of the gravimetric energy density of the bundles in group 3. Here, the effective volume ($V_{eff}$) of the bundle is estimated from $V_{eff}=S_{bd}\times L$, with L representing the sample length. Generally, the bundle with small constituent SWNTs (higher mass density) possesses a much higher volumetric energy density.

**Figure 5:**
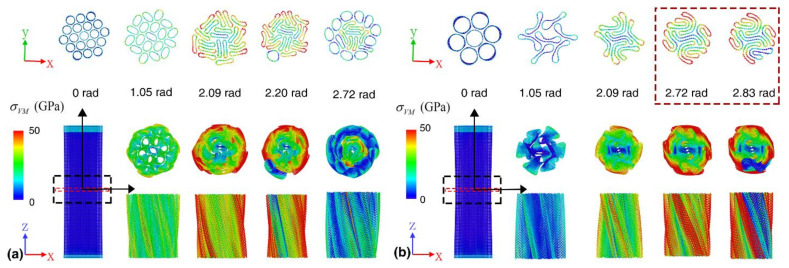
Atomic configurations at different deformation stages for: (**a**) $A_{11}^{19}$ bundle; and (**b**) $A_{20}^{7}$ bundle. Top panels are the cross-sectional views of the bundle in the middle portion (~0.3 nm). Bottom left is the front view of the representative bundles. Bottom right panels are the end-on and side views of a portion of the bundle (~6 nm). Atoms are colored according to the atomic Von Mises (VM) stress.

From the atomic configurations as presented in Figure 5a–b, folding of collapsed wall occurs in the $A_{20}^{7}$ bundle during the deformation, which will affect the associate tensile deformation of the constituent SWNTs. Compared with the fracture strain under pure tension, the equivalent torsion-induced tensile strain ranges from 22.0% to 36.5% for the constituent SWNTs. The folding process of the collapsed CNTs of $A_{20}^{7}$ bundle leads to the highest gravimetric energy density and elastic constant in the three groups. Supporting Information Figure S7 gives the coil radius of each filament in the bundles before fracture. The relative difference between the maximum and minimum coil radius is about 14%, 30% and 34% for $A_{5}^{19}$, $A_{8}^{19}$ and $A_{11}^{19}$ bundle, respectively. During the torsional deformation, the resulting radial compression will reduce the coil radius. The coil radius of the outer CNTs for the bundle with 19 CNTs is generally higher than that of the bundle with seven CNTs. Such observation indicates promoted tensile components in the bundle structure with smaller constituent SWNTs. Overall, these results further affirm that the bundle structure with the same effective diameter can exhibit a vastly different torsional performance.

### 3.5. Bundle Structure Consists of Double-Walled CNTs with Similar Diameter

**Figure 6:**
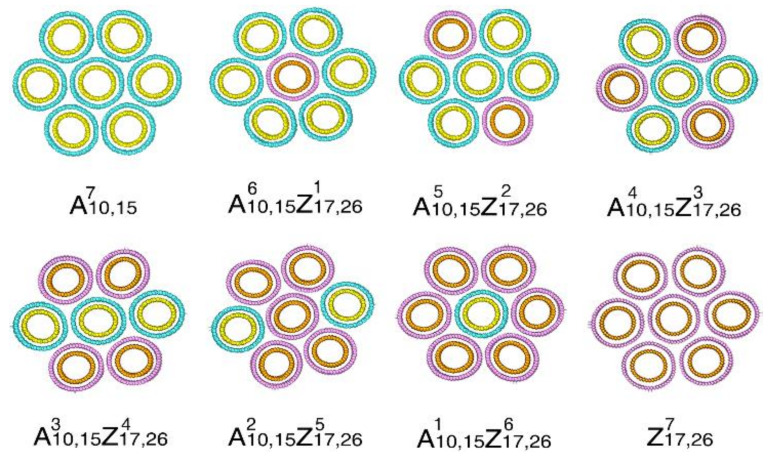
Typical cross-sectional snapshots of DWNT bundles: armchair CNTs are mixing with different ratios of zigzag CNTs, only considering axisymmetric structures.

Given the above impacts on the torsional properties of CNT bundles from different constituent SWNTs, it is of great interest to explore the bundle structures with different double-walled CNTs (DWNTs). For such purpose, we consider the bundle structures constructed from a combination of armchair (10,10)@(15,15) and zigzag (17,0)@(26,0) DWNTs, whose diameters are similar (20.35±0.01 Å). They are denoted as $A_{10,15}$ and $Z_{17,26}$, respectively. To reduce the model size, we consider the bundle structure with seven DWNTs. Starting from the bundle constructed from seven $A_{10,15}$ DWNTs and eight different bundles are obtained by gradually replacing DWNT $A_{10,15}$ with DWNT $Z_{17,26}$(Table 5). The cross-sectional views of different models are presented in Figure 6. For discussion convenience, these models are denoted as $A_{10,15}^{i}Z_{17,26}^{j}$ with i and j representing the number of the corresponding DWNTs. According to Table 5, the elastic constant decrease linearly when the number of DWNT $Z_{17,26}$ increases. The gravimetric energy density shows a different changing pattern, which decreases until the number of DWNT $Z_{17,26}$ reaches 5, afterward, it increases again. It is worthy to mention that the major difference between the bundles constructed from SWNTs and DWNTs is their mass density. It is obvious that the bundle consisting of DWNTs has a much higher volumetric energy density than its counterpart consisting of SWNTs. The volumetric energy density is between 1240 and 1395$\;\mathrm{kJ}/m^{3}$ for DWNTs-based bundles, which is 48% larger than the SWNTs-based bundles in Table 4.

**Figure 7:**
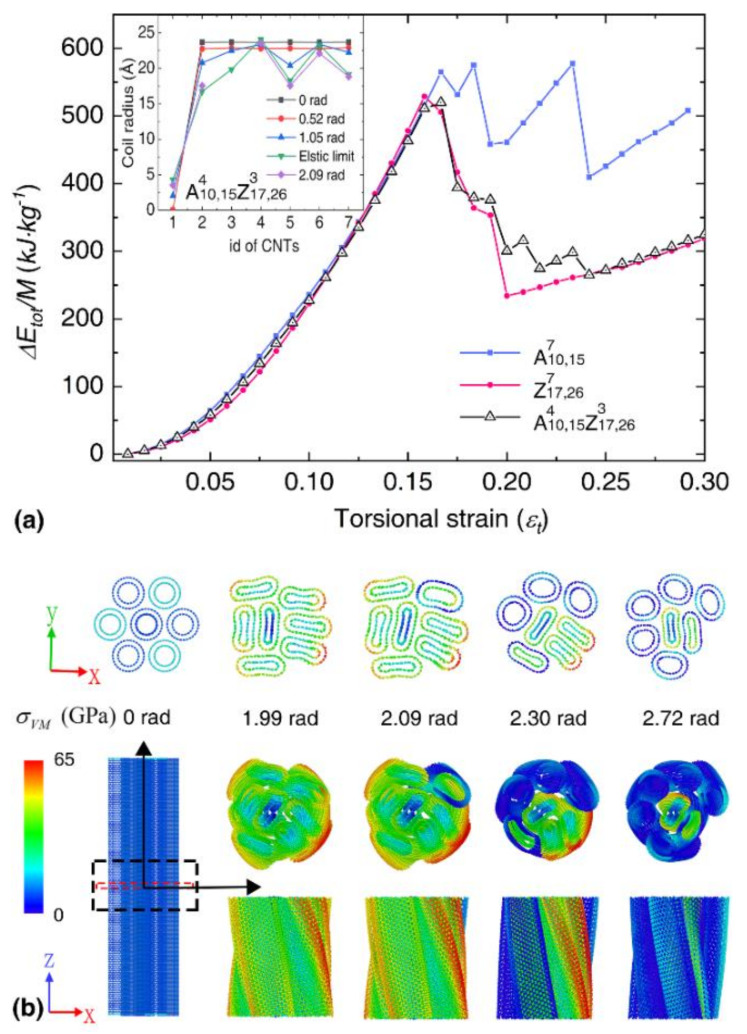
The torsional deformation of bundles with seven constituent DWNTs. (**a**) The strain energy density as a function of the dimensionless torsional strain. Inset shows the change of coil radius for each DWNT in $A_{10,15}^{4}Z_{17,26}^{3}$ bundle under torsion; (**b**) Atomic configurations of $A_{10,15}^{4}Z_{17,26}^{3}$ bundle at different deformation stages. Top panels are the cross-sectional views of the bundle in the middle portion (~0.3 nm). Bottom left is the front view of the representative bundles. Bottom right panels are the end-on and side views of a portion of the bundle (~6 nm). Atoms are colored according to the atomic Von Mises (VM) stress.

Figure 7a compares the strain energy profiles between the representative bundles. A slight mismatch is observed between the strain energy profiles before fracture, indicating the difference of elastic constants between different bundles. After the fracture, multiple strain energy drop events are observed, which is similar to the bundles consisting of SWNTs. As illustrated in Figure 7b, stress concentration is observed at the circumference of the bundle $A_{10,15}^{4}Z_{17,26}^{3}$, and the surface DWNTs experience a fracture at the end region due to its fixed boundary condition. Note, that separate fractures of the inner and outer walls of DWNTs are observed, which are also shown in other kinds of hybrid bundles, as illustrated in Supporting Information Figure S8.

**Figure 8:**
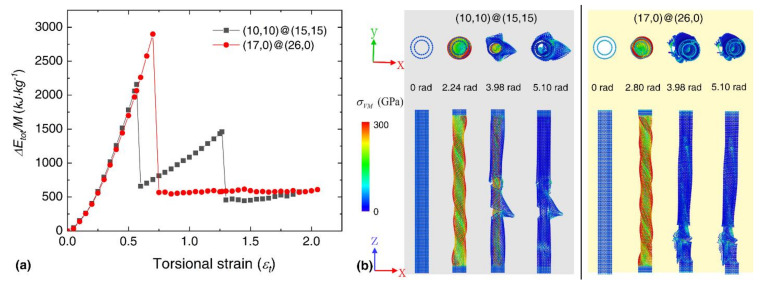
(**a**) The strain energy density as a function of the dimensionless torsional strain; Atomic configurations at different deformation stages for (**b**) (10,10)@(15,15) and (17,0)@(26,0) DWNT; Atoms are colored according to the atomic Von Mises (VM) stress, upper panels are the end-on views, and bottom panels are the side views; The bottom and top fixed edges are treated as a rigid body.

In order to identify the factors that dominate the fracture mode of DWNT-based bundles, we investigated the atomic configurations of individual DWNTs at different torsional deformation stages. Unlike the SWNTs, the individual $A_{10,15}$ DWNT experiences two sudden strain energy drop events during torsion, which is caused by the separate fracture of the outer and inner wall (see Figure 8). Its gravimetric energy density is about 2156.77 kJ/kg, with an elastic limit of 0.57. In comparison, the $Z_{17,26}$ DWNT exhibits a single strain energy drop, as the failure is found to occur simultaneously at the outer and inner walls, which results in a higher gravimetric energy density of about 2899.14 kJ/kg ($\varepsilon_{lim}=\sim$0.70). The torsional strain is illustrated by $\varepsilon_{t}=\varphi D_{0}/l_{0}$, with $D_{0}$ and $l_{0}$ as the equivalent diameter and length of CNT, and φ as the twist angle, it is obvious the outer wall (with larger diameter) experiences earlier fracture than the inner wall. And when the inner and outer shells of armchair@amchair and zigzag@zigzag satisfy the AB stacking of graphite [17,61], the interactional energies or frictions between the inner and outer shells are much larger than the other stacking counterparts [61,62]. In this case, the chirality has a great influence on the torsional mechanical properties of DWNT-based bundles.

**Figure 9:**
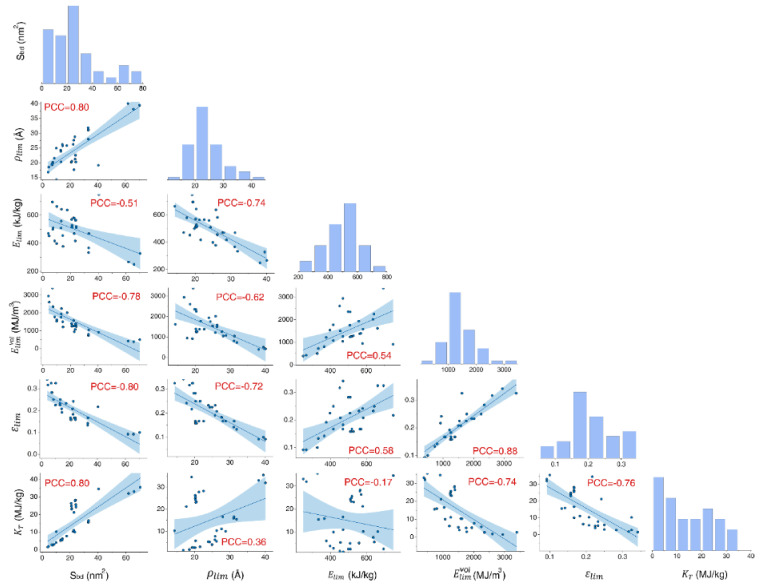
Pairwise data relationship between the gravimetric energy density ($E_{lim}$), elastic limit ($\varepsilon_{lim}$), elastic constant ($k_{T}$), maximum coil radius ($\rho_{lim}$) under the elastic limit stage, volumetric energy density ($E_{lim}^{vol}$) and the cross-sectional area ($S_{bd}$) of CNT-based bundles; together with the relative frequency of each performance parameter. Here, PCC refers to the Pearson correlation coefficient.

Before concluding, we compare the torsional properties of all investigated bundles. The Pearson correlation coefficient (PCC) is calculated, which measures the linear correlation between two variables. It has a value between +1 and −1, where +1, 0 and −1 denote positive, none, and negative linear correlation, respectively. According to Figure 9, the maximum coil radius ($\rho_{lim}$) and the elastic constant ($k_{T}$) are positively related with the cross-sectional area ($S_{bd}$), while the gravimetric energy density ($E_{lim}$) and elastic limit ($\varepsilon_{lim}$) shows a negative relation with the cross-sectional area. A larger elastic limit ($\varepsilon_{lim}$) usually leads to higher volumetric energy density ($E_{lim}^{vol}$), such a positive linear relationship is weakened for the gravimetric energy density ($E_{lim}$). A remarkable reduction of the elastic constant is observed when the elastic limit increases from 0.10 to 0.325. Overall, these results indicate that the packing morphologies and geometry characteristics will significantly affect the torsional behaviors of the CNT bundles.

## 4. Conclusions

Based on atomistic simulations, this work systematically investigates the torsional properties of CNT bundles with different constituent CNTs. Generally, the bundles exhibit a multiple-step fracture behavior due to the uneven torsion-induced tensile stress, i.e., the outer CNTs experience fracture first. The chirality of CNTs, diameter ratio of the constituent CNTs and the mass density of the bundle structures induce significant influence on the torsional properties of the bundle. For a similar diameter, the bundle structure consists of pure zigzag SWNTs usually have a smaller gravimetric energy density and fracture strain (or elastic limit) than its counterpart consisting of pure armchair SWNTs. It is found that a larger diameter ratio exerts a negative effect on the gravimetric energy density (elastic limit) of the bundle structure, but it enhances the elastic constant. The different packing morphologies of the bundle structure with the same components induce a 30% difference to the gravimetric energy density, a 16% and 19% difference to elastic limit and elastic constants, respectively. Specifically, the elastic constant increases as the effective diameter of bundle structures rise, and the relative difference of the elastic constant for the bundle with different mass density decreases from 73% to 53%. Additionally, separate fracture of the outer and inner wall is found in the DWNT-based bundles, resulting in lower gravimetric energy density and elastic limit than those of the SWNT-based bundles. In summary, this work provides a comprehensive understanding of the impacts of microstructure on the torsional properties of CNT bundles.

## Figures and Tables

**Figure 3:**
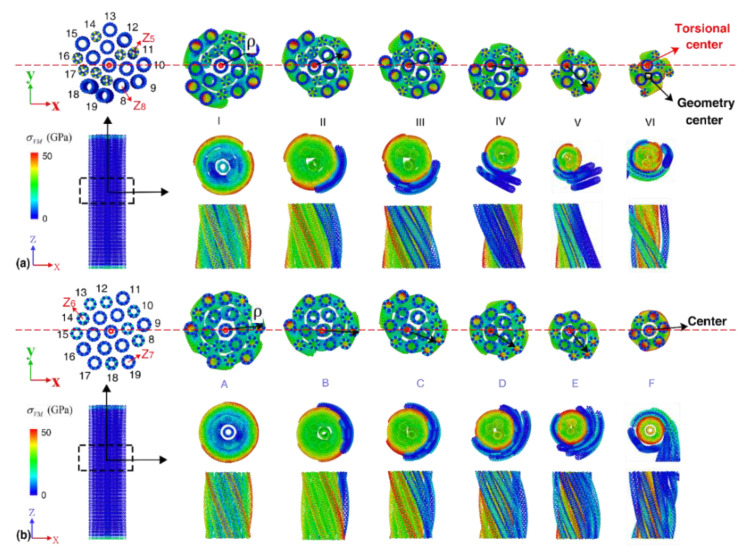
The atomic configurations at different deformation stages for: $A_{5}^{7}Z_{8}^{12}$ bundle (**a**) and $A_{6}^{8}Z_{7}^{11}$ bundle (**b**). I-VI and A-F correspond to the torsional deformation stages of strain energy density curves in Supporting Information Figure S4d. Top panels only show a segment (~6 nm) that close to the fracture region of the sample, and only unfractured CNTs are visualized. Bottom panels are the cross-sectional views of the bundle in the middle portion of the bundle (~6 nm): Bottom left is the front view of the representative bundles; Bottom right panels are the end-on and side views of the middle section of the bundle. Atoms are colored according to the atomic Von Mises (VM) stress.

**Table 1:** Torsional properties of SWNTs with different diameters ($d_{ins}$), including the gravimetric energy density ($E_{lim}$), elastic limit ($\varepsilon_{lim}$), and the elastic constant ($k_{T}$). Here, A and Z represent the armchair or zigzag constituent CNTs, respectively. The subscript represents the index number of the corresponding CNT. N refers to the total atom number for a sample length (L) of 25 nm.

Notation	(m,n)	N	L (Å)	$d_{ins}\;(Å)$	$E_{lim}$ (MJ/kg)	$\varepsilon_{lim}$	$k_{T}$ (MJ/kg)
A_5_	(5,5)	2030	247.96	6.78	2.83	1.40	3.07
A_7_	(7,7)	2842	247.96	9.49	3.15	1.43	5.40
A_8_	(8,8)	3248	247.96	10.85	3.03	1.43	6.70
A_9_	(9,9)	3654	247.96	12.20	2.79	1.38	8.32
A_10_	(10,10)	4060	247.96	13.56	2.64	1.32	10.16
A_11_	(11,11)	4466	247.96	14.92	2.43	1.25	12.24
A_15_	(15,15)	6090	247.96	20.34	2.16	1.04	13.28
A_16_	(16,16)	6496	247.96	21.70	2.23	1.02	12.45
A_20_	(20,20)	8120	247.96	27.12	2.50	0.97	9.31
Z_5_	(5,0)	1145	242.38	3.91	3.93	1.87	1.47
Z_6_	(6,0)	1374	242.38	4.70	1.99	1.12	1.87
Z_7_	(7,0)	1603	242.38	5.48	2.83	1.34	2.26
Z_8_	(8,0)	1832	242.38	6.26	2.92	1.37	2.65
Z_9_	(9,0)	2061	242.38	7.05	2.94	1.36	3.12
Z_10_	(10,0)	2290	242.38	7.83	2.63	1.24	3.62
Z_12_	(12,0)	2748	242.38	9.39	2.96	1.33	4.86
Z_13_	(13,0)	2977	242.38	10.18	3.03	1.38	5.69
Z_15_	(15,0)	3435	242.38	11.74	2.84	1.34	7.49
Z_16_	(16,0)	3664	242.38	12.53	2.80	1.33	8.44
Z_19_	(19,0)	4351	242.38	14.87	2.68	1.27	11.94
Z_26_	(26,0)	5954	242.38	20.36	2.24	1.02	13.36

**Table 2:** Torsional properties for bundles consist of different SWNTs with a similar diameter ($d_{ins}$), including the gravimetric energy density ($E_{lim}$), elastic limit ($\varepsilon_{lim}$), and the elastic constant ($k_{T}$). Here, A and Z represent the armchair or zigzag constituent CNTs, respectively. The subscript and superscript represent the index number and the number of the corresponding constituent CNTs, respectively. $D_{bd}$, η and $S_{bd}$ represent the effective diameter, diameter ratio and the cross-sectional area of the bundle structures, respectively. $\rho_{lim}$ is the maximum coil radius at the elastic limit for outer CNTs in each bundle.$\;N_{b}$ refers to the total atom number for the bundle structures that length (L) of 25 nm.

Group	Notation	$N_{b}$	$D_{bd}$ (nm)	η	$\rho_{lim}$ (Å)	$S_{bd}$ (nm^2^)	$E_{lim}$ (kj/kg)	$\varepsilon_{lim}$	$k_{T}$ (MJ/kg)
1	$A_{5}^{19}$	38,570	4.75	1	19.31	6.86	694.09	0.325	2.72
	$Z_{9}^{19}$	39,159	4.88	1	20.16	7.42	510.46	0.283	2.66
	$A_{5}^{8}Z_{9}^{11}$	38,911	4.82	1.04	19.76	7.18	499.18	0.283	2.67
2	$A_{7}^{19}$	53,998	6.11	1	20.29	13.44	642.94	0.250	5.72
	$Z_{12}^{19}$	52,212	6.06	1	24.30	13.16	508.77	0.225	5.17
	$A_{7}^{8}Z_{12}^{11}$	53,360	6.06	1.01	23.88	13.28	558.18	0.233	5.29
3	$A_{9}^{19}$	69,426	7.46	1	26.25	22.21	581.55	0.208	10.32
	$Z_{16}^{19}$	69,616	7.62	1	28.79	23.43	416.64	0.167	10.03
	$A_{9}^{13}Z_{16}^{6}$	69,486	7.54	1.03	27.64	22.59	469.85	0.183	11.07
4	$A_{11}^{19}$	84,854	8.82	1	27.99	33.22	469.25	0.167	16.57
	$Z_{19}^{19}$	82,669	8.88	1	31.89	33.00	333.94	0.133	15.52
	$A_{11}^{7}Z_{19}^{12}$	83,474	8.81	1.00	31.07	33.08	366.61	0.142	15.89
5	$A_{16}^{19}$	123,424	12.21	1	39.49	70.27	327.95	0.100	35.53
	$Z_{26}^{19}$	113,126	11.54	1	40.09	61.86	266.80	0.092	31.98
	$A_{16}^{9}Z_{26}^{10}$	118,004	11.87	1.07	38.16	65.84	249.15	0.092	33.10

**Table 3:** Torsional properties for bundles consist of zigzag SWNTs with a different diameter ratio (η), including the gravimetric energy density ($E_{lim}$), elastic limit ($\varepsilon_{lim}$), and the elastic constant ($k_{T}$). Here, Z represents the zigzag constituent CNTs. The subscript and superscript represent the index number and the number of the corresponding constituent CNTs, respectively. $D_{bd}$, and $S_{bd}$ represent the effective diameter, and the cross-sectional area of the bundle structures, respectively.$\;\rho_{lim}$ is the maximum coil radius at the elastic limit for outer CNTs in each bundle. $N_{b}$ refers to the total atom number for the bundle structures that length (L) of 25 nm.

Group	Notation	$N_{b}$	η	$D_{bd}$ (nm)	$S_{bd}$ (nm^2^)	$\rho_{lim}$ (Å)	$E_{lim}$ (kj/kg)	$\varepsilon_{lim}$	$k_{T}$ (MJ/kg)
1	$Z_{5}^{7}Z_{8}^{12}$	29,999	1.60	3.90	4.53	18.52	451.87	0.317	1.77
	$Z_{6}^{8}Z_{7}^{11}$	28,625	1.17	3.90	3.98	16.79	470.21	0.342	1.47
2	$Z_{7}^{7}Z_{12}^{12}$	44,197	1.71	5.08	9.96	24.95	398.07	0.225	3.99
	$Z_{9}^{8}Z_{10}^{11}$	41,678	1.11	5.08	8.51	21.54	416.69	0.250	3.01
3	$Z_{10}^{10}Z_{15}^{9}$	53,815	1.50	6.25	14.56	25.75	377.66	0.192	6.22
	$Z_{12}^{9}Z_{13}^{10}$	54,502	1.08	6.25	14.37	26.21	455.50	0.208	5.51

**Table 4:** Torsional properties for bundles containing the same constituent CNTs, including the gravimetric energy density ($E_{lim}$), volumetric energy density ($E_{lim}^{vol}$), elastic limit ($\varepsilon_{lim}$), and the elastic constant ($k_{T}$). Here, A represents the armchair constituent CNTs. The subscript and superscript represent the index number and the number of the corresponding constituent CNTs, respectively. $D_{bd}$, and $S_{bd}$ represent the effective diameter, and the cross-sectional area of the bundle structures, respectively. N is the total atom number.$\;\rho_{lim}$ is the maximum coil radius at the elastic limit for outer CNTs in each bundle. $N_{b}$ refers to the total atom number for the bundle structures that length (L) of 25 nm.

Group	Notation	$N_{b}$	$D_{bd}$ (nm)	$\rho_{lim}$ (Å)	$E_{lim}$ (kj/kg)	$E_{lim}^{vol}$ $\left(\mathrm{MJ}/m^{3}\right)$	$\varepsilon_{lim}$	$k_{T}$ (MJ/kg)
1	$A_{5}^{19}$	38,570	4.75	19.46	694.09	3396	0.325	2.72
	$A_{10}^{7}$	28,420	4.75	14.28	662.15	1620	0.325	10.25
2	$A_{8}^{19}$	61,712	6.78	25.82	637.04	2016	0.233	7.74
	$A_{15}^{7}$	42,630	6.78	17.66	579.06	944	0.242	21.22
3	$A_{11}^{19}$	84,854	8.82	31.18	469.25	1043	0.167	16.57
	$A_{20}^{7}$	56,840	8.82	19.16	748.19	915	0.217	34.58

**Table 5:** Torsional properties for bundles consisting of DWNTs, including the gravimetric energy density ($E_{lim}$), elastic limit ($\varepsilon_{lim}$), and the elastic constant ($k_{T}$). Here, A and Z represent the armchair and zigzag constituent DWNTs. The subscript and superscript represent the index number and the number of the corresponding constituent DWNTs, respectively. $D_{bd}$ and $S_{bd}$ represent the effective diameter, and the cross-sectional area of the bundle structures, respectively. N is the total atom number. $\rho_{lim}$ is the maximum coil radius at the elastic limit for outer CNTs in each bundle. $N_{b}$ refers to the total atom number for the bundle structures that length (L) of 25 nm.

Notation	$N_{b}$	$S_{bd}\;({\mathrm{nm}}^{2})$	$\rho_{lim}\;(\text{Å})$	$E_{lim}\;(\mathrm{kJ}\mathrm{kg})$	$E_{lim}^{vol}$ $\;(\mathrm{MJ}/m^{3})$	$\varepsilon_{lim}$	$k_{T}$ (MJ/kg)
$A_{10,15}^{7}$	71,400	23.87	22.55	565.08	1380	0.167	28.28
$A_{10,15}^{6}Z_{17,26}^{1}$	71,348	23.58	21.22	562.92	1378	0.167	27.85
$A_{10,15}^{5}Z_{17,26}^{2}$	71,296	20.77	20.17	569.74	1395	0.167	26.35
$A_{10,15}^{4}Z_{17,26}^{3}$	71,244	24.10	20.23	519.18	1262	0.167	25.64
$A_{10,15}^{3}Z_{17,26}^{4}$	71,192	20.91	20.20	515.97	1254	0.158	24.85
$A_{10,15}^{2}Z_{17,26}^{5}$	71,140	24.21	20.26	509.85	1240	0.158	24.30
$A_{10,15}^{1}Z_{17,26}^{6}$	71,088	21.22	20.52	514.58	1254	0.158	23.34
$Z_{17,26}^{7}$	71,036	21.06	20.35	528.33	1288	0.158	23.08

## Data Availability

The data that support the findings of this study are available from the corresponding authors on reasonable request.

## Supplementary Materials

The following supporting information can be downloaded at: https://www.mdpi.com/article/10.3390/nano12050760/s1. The Supporting Information is available free of charge, including: Torsional strain energy curves and atomic configurations of SWNTs in Supporting Information Figure S1; Torsional strain energy density vs. the dimensionless torsional strain of the representative SWNTs in Supporting Information Figure S2; Coil radius of the outer CNTs and effective diameter of bundles consists of SWNTs with different chirality in Supporting Information Figure S3; Torsional deformation and strain energy curves for SWNT bundles with different diameter ratio in Supporting Information Figure S4; Torsional properties and geometry characteristics for bundles with different packing modes in Supporting Information Figure S5; The torsional strain energy density as a function of the dimensionless torsional strain for bundles with different mass density in in Supporting Information Figure S6; Geometry characteristics for SWNT bundles with different mass density in Supporting Information Figure S7; Coil radius of each filament under different deformation stages for representative double-walled CNT bundles in Supporting Information Figure S8.
