# Supplementary material for: Torsional Properties of Bundles with Randomly Packed Carbon Nanotubes
Source: Nanomaterials (Basel). 2022 Feb 24;12(5):760. doi: 10.3390/nano12050760 (PMC8911843; doi:10.3390/nano12050760)
Supplement: Supplementary file 1 [file nanomaterials-12-00760-s001.zip › nanomaterials-1585881-supplementary.pdf]

Supplementary Information

Torsional Properties of Bundles with Randomly Packed Carbon Nanotubes

Hanqing Wei[1], Heidi Zhi Jin Ting[2], Yongji Gong[3], Chaofeng Lü[1,4], Olga E. Glukhova[5,6], and Haifei Zhan[1,2,*]

[1]*Department of Civil Engineering, Zhejiang University, Hangzhou 310058, China*

[2]*School of Mechanical, Medical and Process Engineering, Queensland University of Technology (QUT), Brisbane, QLD 4001, Australia*

[3]*School of Materials Science and Engineering, Beihang University, Beijing 100191, China*

[4]*Soft Matter Research Center, Zhejiang University, Hangzhou 310027, China*

[5]*Department of Physics, Saratov State University, Astrakhanskaya str. 83, Saratov 410012, Russia*

[6]*Institute for Bionic Technologies and Engineering, I.M. Sechenov First Moscow State Medical University (Sechenov University), 119991, Russia*

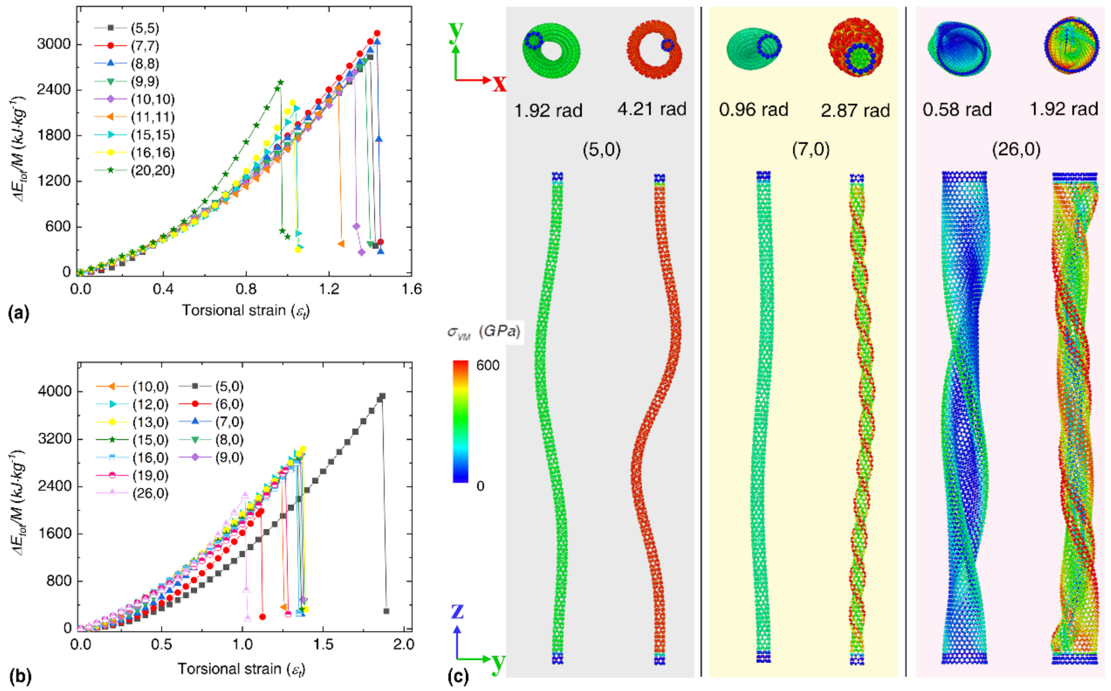

Supplementary Figure S1 | Torsional strain energy curves and atomic configurations of SWNTs. Torsional strain energy density vs. dimensionless torsional strain for armchair (a) and zigzag (b) SWNTs. (c) The corresponding atomic configurations of (5,0), (7,0) and (26,0) SWNTs at different deformation stages. The examined SWNTs exhibit torsional elastic constant ranges from 1.5 to 13.7 MJ/kg, elastic limit is 1.0~1.9, and gravimetric energy density is in the range of 2.0 to 3.15 MJ/kg. The twisting-induced helical buckling, torsional buckling and collapsed wall folds are found in the (5,0), (7,0) and (26,0) SWNTs, respectively. The columns are colored based on the atomic Von Mises (VM) stress. Upper panels are the end-on views, and bottom panels are the side views. The two blue regions of the SWNTs are the fixed edges and treated as rigid body.

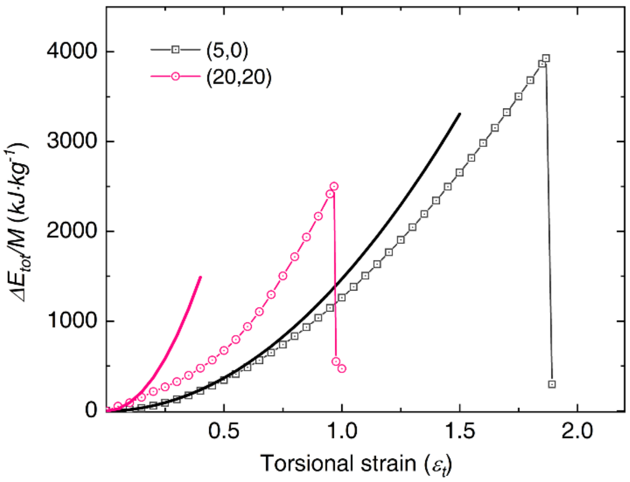

Supplementary Figure S2 | Torsional strain energy density vs. the dimensionless torsional strain of the representative SWNTs. Solid lines are the corresponding fitting curves based on Hooke's law. For the convenience of comparison, the elastic constant is calculated based on ε_t =0.05 for all the SWNTs.

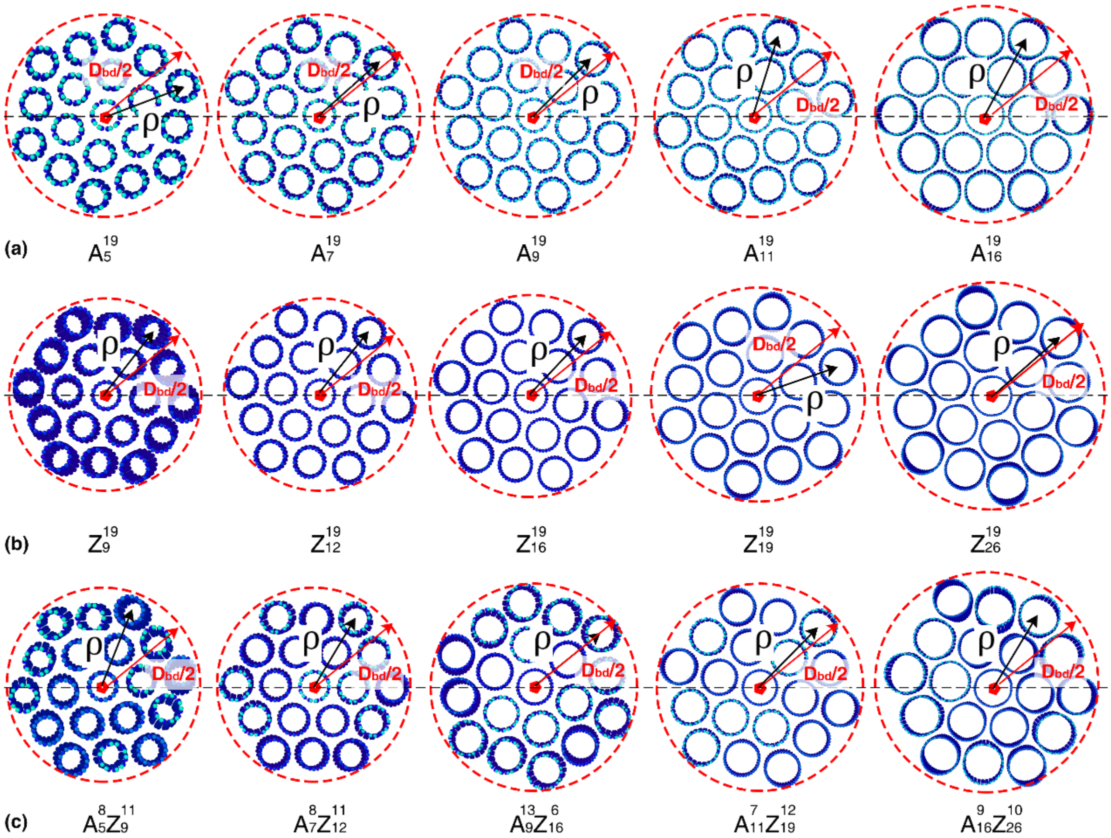

Supplementary Figure S3 | Coil radius and effective diameter of the bundles consisting of: armchair SWNTs (a), zigzag SWNTs (b) and mixed SWNTs (c).

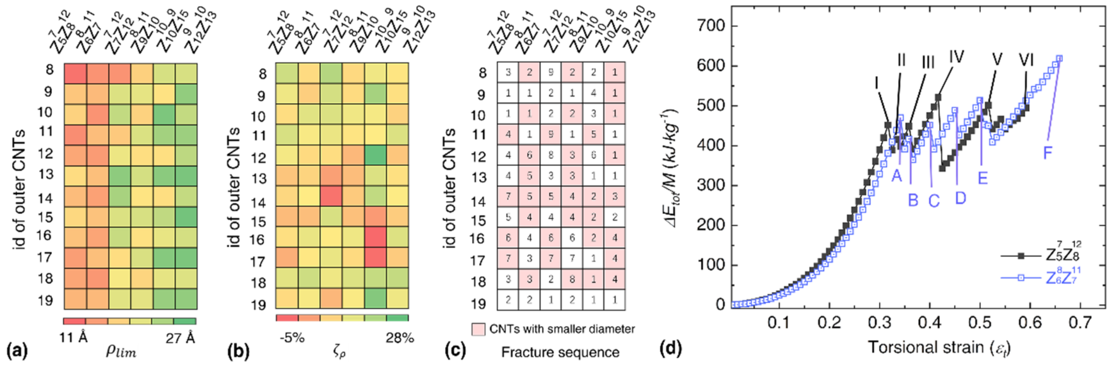

Supplementary Figure S4 | Torsional deformation and strain energy curves for SWNT bundles with different diameter ratio. (a) Coil radius of the outer CNTs at the elastic limit stage for different bundles; (b) The relative difference of coil radius for the outer CNTs between the initial state and the elastic limit stage; (c) The fracture sequence of the outer CNTs. The pink regions indicated the CNTs with smaller diameter and the number in the grid represents the fracture sequence of the constituent CNTs; (d) The torsional strain energy density as a function of the dimensionless torsional strain for $A_5^7Z_8^{12}$ and $A_6^8Z_7^{11}$ bundle.

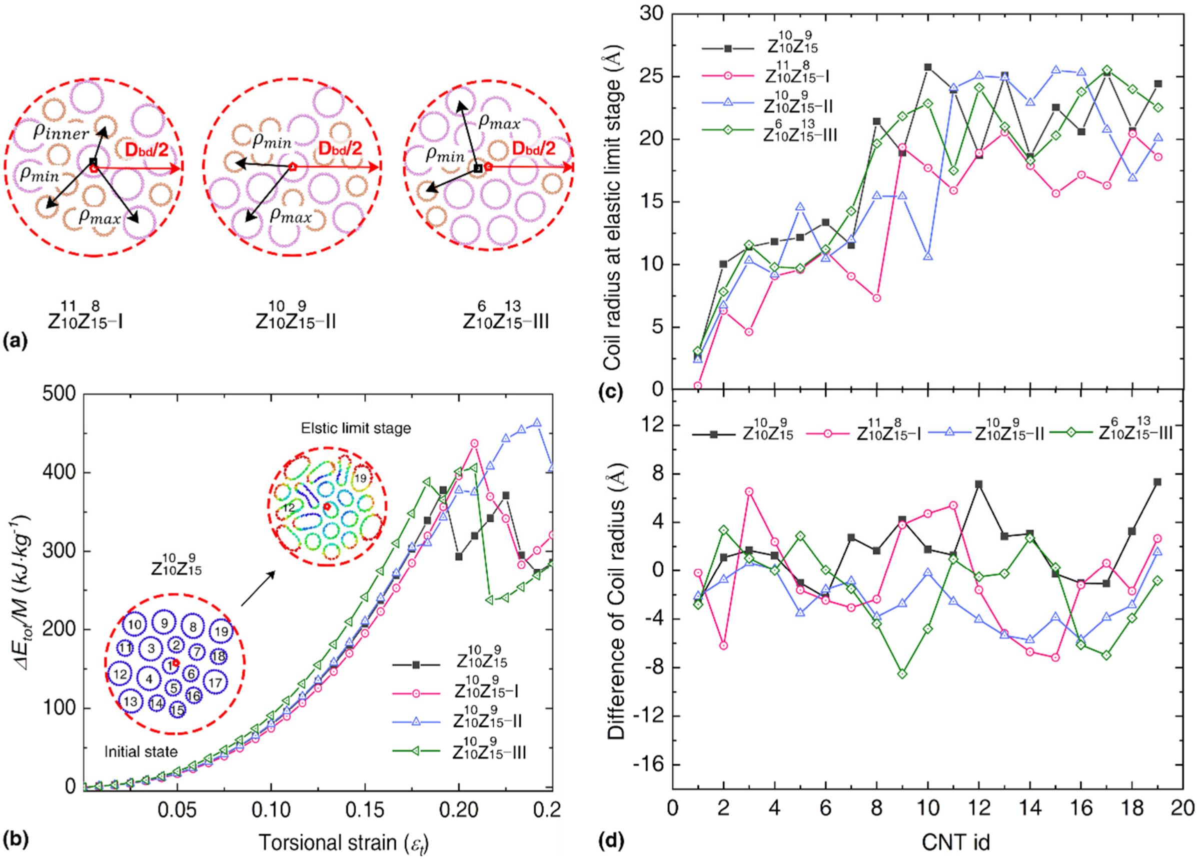

Supplementary Figure S5 | Torsional properties and geometry characteristics for SWNT bundles with different packing modes: (a) The strain energy density as a function of the dimensionless torsional strain for bundle structures with a diameter ratio of 1.5. (b) Coil radius of each CNT at the elastic limit stage (before fracture); From the geometry, the coil radius of the CNT-based bundle can divide into three parts: The coil radius of the outer CNTs (id of CNTs increases from 8 to 12) is 15.5~25.5 Å; The coil radius of the inner CNTs is between 5.0 and 14.5 Å; The coil radius of the center CNT is below 3.0 Å. (c) The boxplot of the coil radius of outer CNTs at the elastic limit. The mean coil radius of outer CNTs is ~20.0 Å. (d) Difference of coil radius between the initial state and elastic limit stage. The relative difference of it changes with the location of the CNTs, with a range of -8.0~8.0 Å.

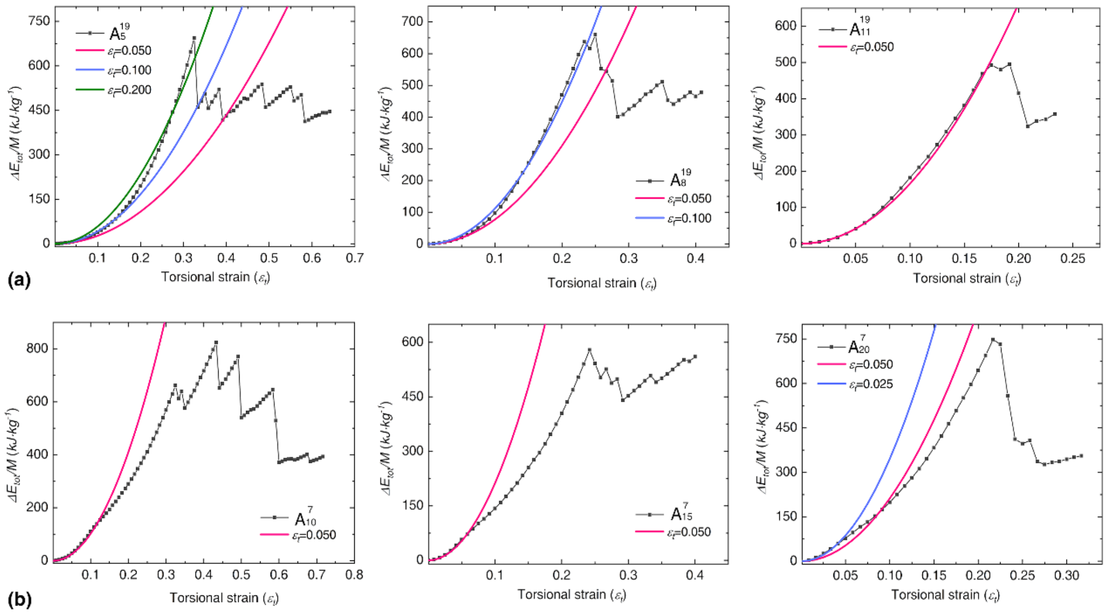

Supplementary Figure S6 | The torsional strain energy density as a function of the dimensionless torsional strain for: bundles with large (19 small CNTs) (a) and small (7 large CNTs) (b) mass density.

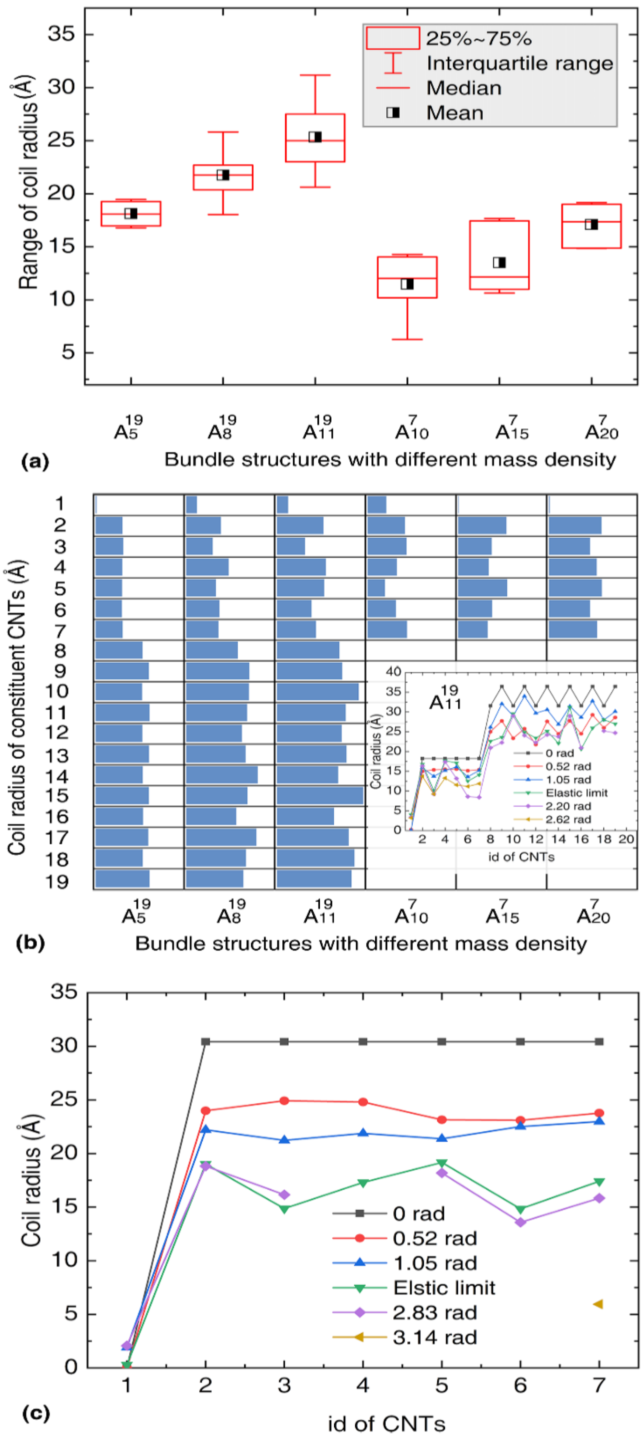

Supplementary Figure S7 | Geometry characteristics for SWNT bundles with different mass density. (a) The boxplot of the coil radius for outer CNTs at the elastic limit stage; (b) Coil radius distribution of the CNTs at the elastic limit, Inset shows the coil radius for A_{11}^{19} bundle under different torsional deformation stages; (c) The coil radius for A_{20}^{7}bundle under different torsional deformation stages; □fter the elastic limit stage, only the coil radius of the intact CNTs are visualized.

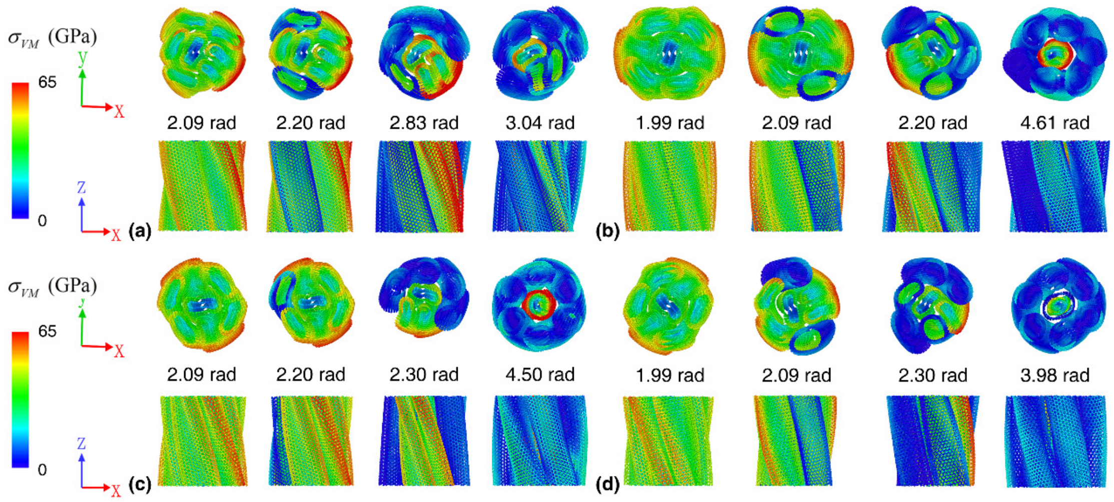

Supplementary Figure S8 | Atomic configurations of bundles: $A^{7}_{10,15}$ (a), $Z^{7}_{17,26}$ (b), $A^{5}_{10,15}Z^{2}_{17,26}$ (c) and $A^{3}_{10,15}Z^{4}_{17,26}$ (d) at different deformation stages. The columns are colored based on the atomic Von Mises (VM) stress. Upper panels are the end-on views and bottom panels are the side views of the segment (~6 nm) of the whole sample.
